# Glucocorticoid receptor complexes form cooperatively with the Hsp90 co-chaperones Pp5 and FKBPs

**DOI:** 10.1038/s41598-020-67645-8

**Published:** 2020-07-01

**Authors:** Anna Kaziales, Katalin Barkovits, Katrin Marcus, Klaus Richter

**Affiliations:** 1 Center for Integrated Protein Science Munich at the Department of Chemistry, Technische Universität München, Lichtenbergstr. 4, 85748 Garching, Germany; 2 Medical Proteome Center, Ruhr University Bochum, Bochum, Germany

**Keywords:** Biochemistry, Biophysics

## Abstract

The function of steroid receptors in the cell depends on the chaperone machinery of Hsp90, as Hsp90 primes steroid receptors for hormone binding and transcriptional activation. Several conserved proteins are known to additionally participate in receptor chaperone assemblies, but the regulation of the process is not understood in detail. Also, it is unknown to what extent the contribution of these cofactors is conserved in other eukaryotes. We here examine the reconstituted *C. elegans* and human chaperone assemblies. We find that the nematode phosphatase PPH-5 and the prolyl isomerase FKB-6 facilitate the formation of glucocorticoid receptor (GR) complexes with Hsp90. Within these complexes, Hsp90 can perform its closing reaction more efficiently. By combining chemical crosslinking and mass spectrometry, we define contact sites within these assemblies. Compared to the nematode Hsp90 system, the human system shows less cooperative client interaction and a stricter requirement for the co-chaperone p23 to complete the closing reaction of GR·Hsp90·Pp5/Fkbp51/Fkbp52 complexes. In both systems, hormone binding to GR is accelerated by Hsp90 alone and in the presence of its cofactors. Our results show that cooperative complex formation and hormone binding patterns are, in many aspects, conserved between the nematode and human systems.

## Introduction

Hsp90 is an ATP-driven molecular machine, highly abundant in the cytosol. It is recruited to client proteins that require energy-intense rearrangements and supports these reactions by performing nucleotide-induced conformational changes^1,2^. These changes lead to the compaction of the Hsp90 dimer and the transient formation of a ring-like structure, where the N-terminal domains are dimerized in addition to the chaperone’s permanent C-terminal dimerization^3^. Only in the bacterial Hsp90 system are these changes performed and controlled by Hsp90 itself. In the eukaryotic systems, client-specific cofactors are present, which tailor the Hsp90 machinery into a client-specific mode and regulate the conformational changes while providing further interaction sites for the clients^4^. This has been investigated for the Hsp90-dependent maturation of protein kinases with the help of the cofactor Cdc37 and for the chaperoning of steroid receptors, where a larger set of cofactors participates^5–7^. Partly, these reactions have been reconstituted with recombinant proteins to identify interaction mechanisms, but mostly addressing the cofactors’ interaction with Hsp90 in the absence of clients.

Hsp90-containing protein complexes were first identified in the 1980s^8,9^. In these studies, steroid hormone receptors, such as the glucocorticoid, mineralocorticoid or progesterone receptors, were isolated from vertebrate cells and the associated proteins were identified and studied by western blot analyses. Hsp90 and several other proteins were detected in these complexes, leading to the identification of Fkbp51, Fkbp52, Cyp40, Hop, p23, Hip and Pp5 as components of steroid hormone receptor complexes^10–14^. The glucocorticoid receptor (GR) complex had early on been described as a 9 S protein complex with an approximate molecular weight of 300,000 Da^15^. It has been found to be inactive in DNA-binding^16–18^ and hormone binding^19^ and its conversion to the hormone-binding state requires the participation of the chaperone machinery^20^. In the end, a hyperphosphorylated^21^ and transcriptionally active GR is transported to the nucleus^22^.

Based on mutated or truncated variants of the involved proteins, binding sites were identified and some first mechanistic details were uncovered^23–29^. The studies finally led to a reaction cycle in which a steroid receptor is first bound by Hsp40, Hsc70 and Hop^30^ and then at later stages by Hsp90, Fkbps and p23^31^. There are interim steps, where ATP-binding and hydrolysis reactions in Hsc70 and Hsp90 induce the conformational changes in the assemblies^21,32–34^. Despite this extensive knowledge, the in vitro assembly and characterization of these complexes remained difficult until recently, when several client proteins became accessible in purified form^35–38^. It is also unclear whether these assemblies are only relevant in vertebrates with steroid receptor signaling or whether other eukaryotes encoding similar forms of ligand-binding receptors also utilize these pathways. In plants, for example, similar Hsp90-containing assemblies have been observed and were later related to the signaling of brassinosteroids^39,40^. For lower eukaryotes, the capabilities and functional conservation of the Hsp90 chaperone system are less clear in this respect.

In a recent study, we investigated the influence of the nematode PPH-5 protein, the TPR-containing phosphatase homolog to human Pp5, on the phosphorylation state of glucocorticoid receptor fragments and observed its dependency on the *C. elegans* Hsp90 (HSP-90)^41^. We see that the dephosphorylation of GR-fragments is enhanced in the presence of HSP-90 and this activity is conserved in the vertebrate system^41,42^. Nevertheless, the role of Pp5 during the assembly of GR·Hsp90 complexes is far from clear. We here set out to understand the mechanistic role of PPH-5 during the formation of HSP-90 containing GR-complexes and investigate the role of other nematode cofactors and their human counterparts in these binding reactions.

## Results

### Open and closed states of GRLBDm complexes with HSP-90 can be formed with the nematode PPH-5

**Figure 1:**
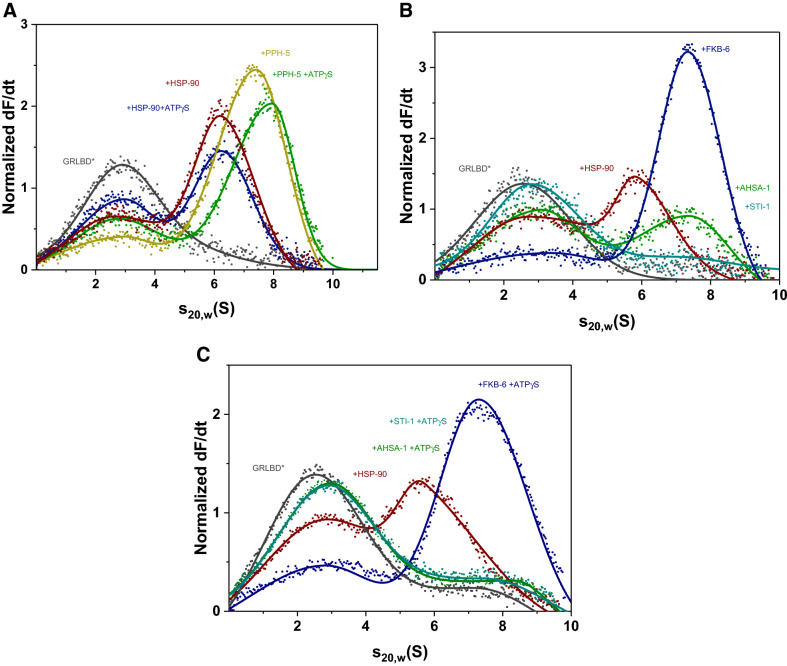
PPH-5 enables the formation of open and closed HSP-90 complexes. (**A**) Sedimentation velocity AUC analysis of *GRLBDm (grey), *GRLBDm ·HSP-90 (red), *GRLBDm·HSP-90/ATPγS (navy), *GRLBDm·HSP-90·PPH-5 (yellow), *GRLBDm·HSP-90/ATPγS·PPH-5 (green) complexes. (**B**) Influence of AHSA-1 (green), STI-1 (petrol) and FKB-6 (navy) cofactors on the formation of *GRLBDm·HSP-90 complexes analyzed by sedimentation velocity AUC. (**C**) The same experimental setup with AHSA-1 (green), STI-1 (petrol) and FKB-6 (navy) was performed in the presence of ATPγS to initiate the closing reaction of HSP-90.

GR-complexes with nematode HSP-90 can accommodate the protein phosphatase PPH-5, which binds via its TPR-domain to HSP-90 and is then active to dephosphorylate the DNA-binding domain of the HSP-90 bound GR^41^. Given that no further information is available on this protein complex, also in other model systems, we were interested to see how the function and affinity are regulated and whether the HSP-90 conformation is restricted. We therefore tested whether the GRLBDm·HSP-90·PPH-5 complex can be influenced by the presence of nucleotides that induce the closed state of HSP-90. To this end, we fluorescently labelled GRLBDm with ATTO 488 and recorded the sedimentation behavior of the protein complexes it is forming by analytical ultracentrifugation (AUC). GRLBDm alone sediments with 2.7 ± 0.2 S, while in the presence of nematode HSP-90 it forms a complex with 6.1 ± 0.2 S. Upon addition of PPH-5 to GRLBDm·HSP-90 a strong shift to higher s_20,w_ with 7.2 S was observed for the ternary complex as reported previously (Fig. 1A)^41^. Furthermore, the addition of PPH-5 led to a significant reduction of free GRLBDm at 2.7 S compared to HSP-90 alone, implying that the presence of PPH-5 increases the affinity of HSP-90 for GRLBDm (Fig. 1A). We then used the slowly hydrolysable ATP analog, ATPγS, to test whether the closing reaction of the nematode Hsp90 protein is possible. The presence of ATPγS indeed leads to an increased sedimentation coefficient (s_20,w_) for the ternary complex from 7.2 S to 7.9 S, implying that GRLBDm·HSP-90·PPH-5 can be influenced by the nucleotide and may become more compact. The increased s_20,w_ matches the behavior of other Hsp90-assemblies, in which the nucleotide-induced closing reaction is observable^43,44^. Despite the increased sedimentation coefficient, the amount of bound GRLBDm decreased and the concentration of free GRLBDm at 2.7 S increased, implying that the ATPγS-induced closing reaction slightly decreases the affinity of the chaperone complex to its client (Fig. 1A). We also tested the nucleotide influence in the absence of PPH-5. Also under these conditions, ATPγS reduces the affinity of HSP-90 for GRLBDm, but the shift representing the closing movement cannot be observed and the s_20,w_ is unchanged at 6.1 S (Fig. 1A). This implies that PPH-5 supports the nucleotide-induced compaction of nematode HSP-90·GRLBDm complexes and simultaneously increases the affinity of the chaperone machine to this client.

### HSP-90 co-chaperones differentially influence GRLBDm complexes

Having observed that PPH-5 forms a complex with HSP-90 and GRLBDm and supports the nucleotide-induced rearrangement of the chaperone, we aimed at testing whether other cofactors of the nematode HSP-90 system are influencing the assembly of the GRLBDm·HSP-90 complex. To this end, we purified the cofactors STI-1, FKB-6 and AHSA-1, all of which had been shown to interact with HSP-90 in the absence of client proteins^45–47^ and to modulate GR activity in the vertebrate or yeast systems^10,11,48^. Addition of STI-1 strongly reduces binding of the GRLBDm to the HSP-90 protein, while AHSA-1 binds in addition, but does not affect the affinity for GRLBDm (Fig. 1B). Interestingly, the addition of FKB-6 leads to a marked increase in affinity and to formation of HSP-90·GRLBDm·FKB-6 ternary complex (Fig. 1B). Thus, the large PPIase FKB-6, like the phosphatase PPH-5, is apparently capable of strengthening the interaction between GRLBDm and the HSP-90 dimer.

We then tested whether these cofactors’ interactions are also sensitive to the nucleotide bound state of the HSP-90 protein, by adding ATPγS to the cofactor containing GRLBDm·HSP-90 complexes (Fig. 1C). This leads to a further diminishing of the complex peak in the case of AHSA-1 and continued depletion in the case of STI-1. In contrast, the FKB-6 containing complex is still intensely formed and its sedimentation coefficient is increased from 6.9 to 7.3 S. This implies that, in the presence of FKB-6, as in the presence of PPH-5, nematode HSP-90 is able to perform its nucleotide-induced rearrangements.

### Cep23/DAF-41 binds to closed GRLBDm·HSP-90·FKB-6/PPH-5 complexes with no influence on the affinity for the client

Given that PPH-5 and FKB-6 support GR complex formation, we aimed at testing how the cofactor Cep23/DAF-41 influences the affinity of the Hsp90 machinery for GRLBDm. The interaction of the Hsp90 machinery with p23 critically depends on nucleotide binding to Hsp90 and the closed conformation of the chaperone^34,49–52^. This interaction reflects a late step during the maturation cycle of GR, at the point where the active hormone binding site is formed^33^.

**Figure 2:**
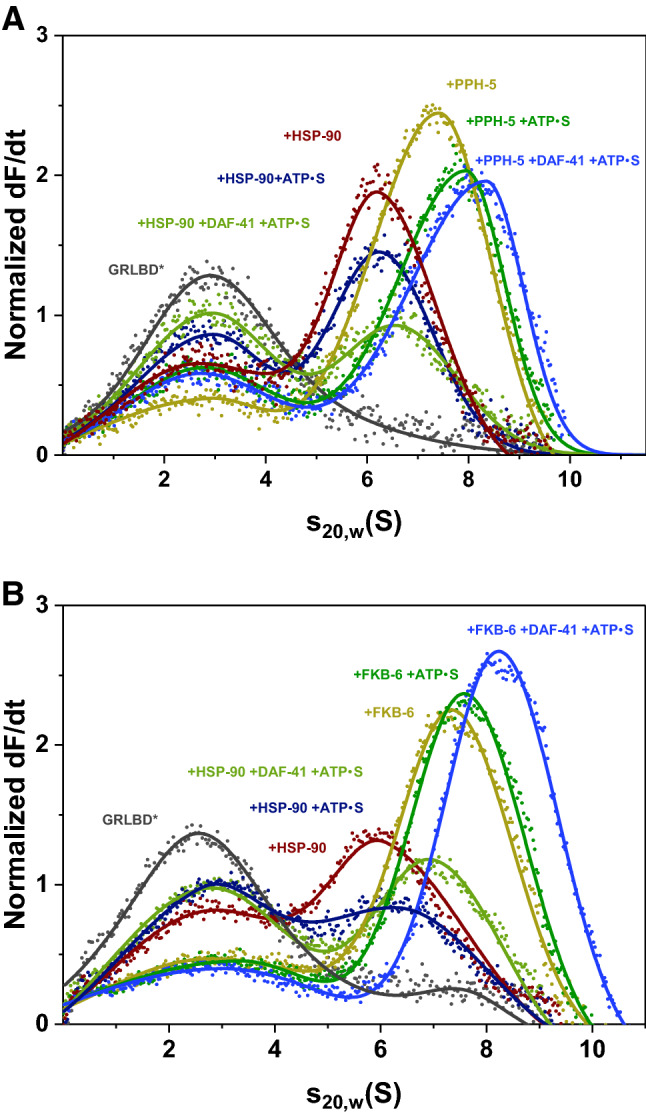
Cep23/DAF-41 can be included in *GRLBDm·HSP-90·PPH-5/FKB-6 complexes. (**A**) Sedimentation velocity AUC analysis of *GRLBDm (grey), *GRLBDm ·HSP-90 (red), *GRLBDm·HSP-90/ATPγS (navy), *GRLBDm·HSP-90/ATPγS·DAF-41 (light green), *GRLBDm·HSP-90·PPH-5 (yellow), *GRLBDm·HSP-90/ATPγS·PPH-5 (green), *GRLBDm·HSP-90/ATPγS·PPH-5·DAF-41 (blue) complexes. (**B**) The same experimental set-up performed accordingly for *GRLBDm·HSP-90 complexes in the presence of FKB-6 and/or Cep23/DAF-41 as described in the plot. In both cases addition of Cep23/DAF-41 leads to an increase in the average sedimentation coefficient of the complex forming species.

AUC experiments were performed to evaluate the effect of DAF-41 in the presence of ATPγS. The ternary GRLBDm HSP-90·DAF-41 complex displayed a reduced affinity for the client compared to HSP-90 alone, as judged from the free GRLBDm fraction (Fig. 2A,B). Binding of GRLBDm to HSP-90·PPH-5 decreased in the presence of ATPγS. The addition of Cep23/DAF-41 did not change this affinity, as almost the same amount of GRLBDm molecules were retained in the chaperone complex. Cep23/DAF-41 binding to the complex can nevertheless be seen from the significant increase in the complex’s sedimentation coefficient towards 8.3 S (Fig. 2A). This was also tested for the PPIase FKB-6 (Fig. 2B). Here, as well, DAF-41 bound in addition, while maintaining the affinity for GRLBDm. It therefore seems that Cep23/DAF-41 has a similar effect on both TPR-cofactor complexes, not influencing the affinity for the client further than the TPR cofactor itself, while interacting with the closed HSP-90 conformation.

### Insight into ternary complex topology

**Figure 3:**
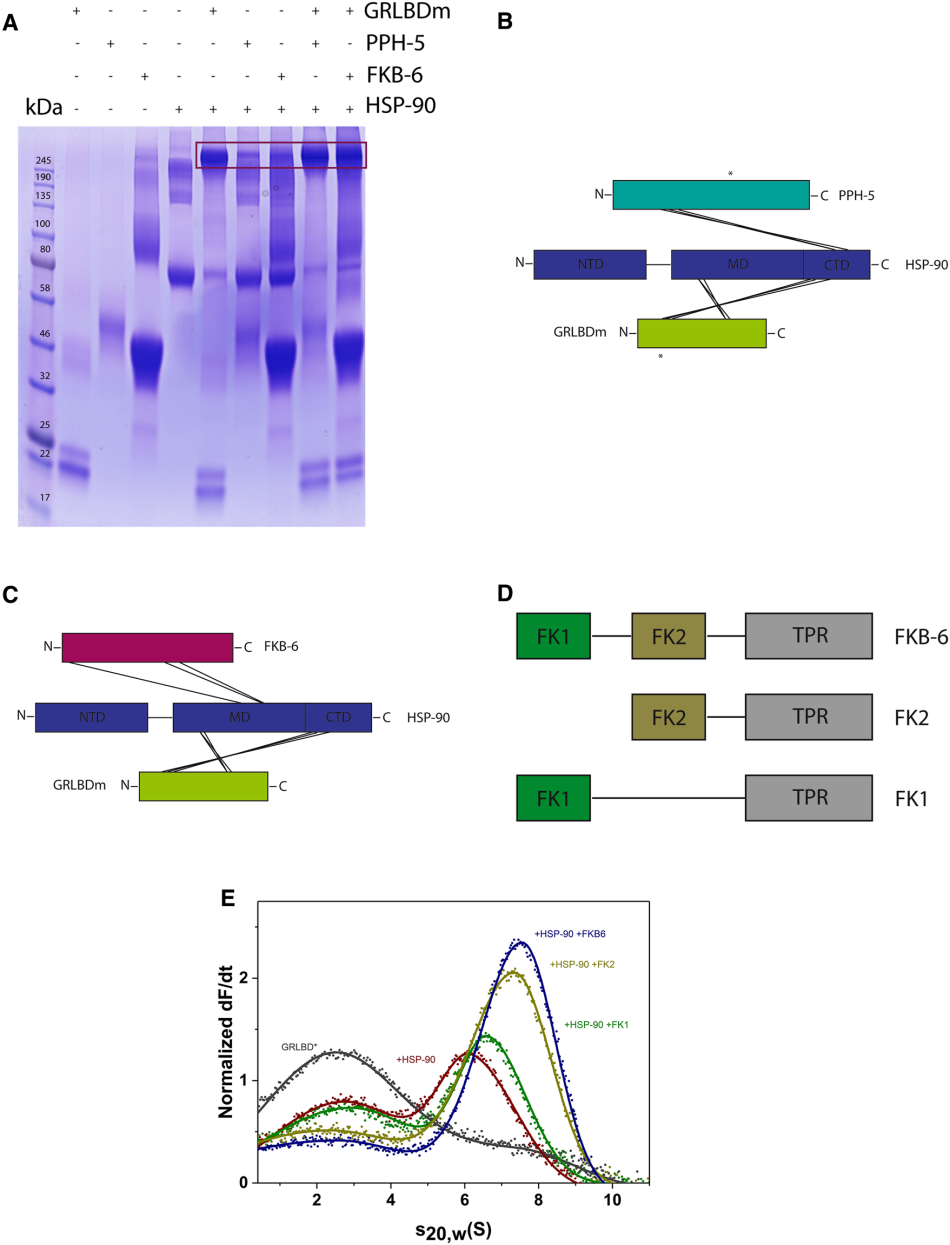
Identification of binding interfaces of GRLBDm· HSP-90·PPH-5/FKB-6 complexes derived from XL-MS. (**A**) Crosslinked protein complexes with composition as indicated in the figure were analysed by SDS-PAGE. Binary or ternary complex representing bands, as highlighted by the frame, were excised and analysed by mass spectrometry. The uncut gel image is shown in Supplementary Fig. S1. (**B**) Schematic representation of the inter-protein crosslinked products detected between HSP-90, GRLBDm and PPH-5. Lines represent the contacts between the two proteins and the chaperone whereas asterisks represent the crosslinked products detected between PPH-5 and GRLBDm. (**C**) Schematic representation of the inter-protein crosslinked products detected between HSP-90, GRLBDm and FKB-6. **(D**) Schematic representation of the full length and truncated FKB-6 variants. E) Domain specific binding of FKB-6 and FKB-6 deletion constructs to GR-LBDm·HSP-90 analysed by sedimentation velocity AUC.

To determine a representative topology of the ternary GRLBDm·HSP-90 complexes with PPH-5 and FKB-6, we searched for binding interfaces by chemical crosslinking and mass spectrometry. The protein complexes were treated with the isotopically labelled crosslinker H_12_/D_12_-BS^3^ and analysed by SDS-PAGE. The complex-representing bands (Fig. 3A) were subjected to tryptic digestion, followed by high resolution mass spectrometry. Our analysis, carried out independently by the in-house software xMASS and pLink, aimed at detecting inter-protein crosslinked peptides, reporting on potential contact sites of the proteins^41,53^.

Our data suggests that GRLBDm is positioned in the middle (M) domain of HSP-90, as we identified peptides of GRLBDm crosslinked to the lysines 326, 329 of HSP-90 (Tables 1, 2). Additional contacts were detected, linking GRLBDm to the C-terminal dimerization domain (CTD) at positions 551,555 and 601. These crosslinking sites were identified in both the PPH-5 (Table 1) and FKB-6 complexes (Table 2).

**Table 1:** Inter-protein crosslinked peptides detected for GRLBDm·HSP-90·PPH-5 complexes.

Peptide 1	Peptide 2	Lys 1	Lys 2	MS2 scan	Hit number in scan	Hit % spectral intensity	Independent analysis
**Peptide HSP-90**	**Peptide GR-LBD**						
APFDLFENKK	ELGKAIAK	326	699	24,061	30	99	✓
DSSTMGYMAAKK	QVIAAVKWAK	601	576	22,378	84	92	✓
APFDLFENKK	MTYIKELGK	326	695	27,214	84	88	✓
SKNSIK	MTYIKELGK	329	695	10,548	29	99	✓
VEKVGVSNR	WAKAIPGFR	555	579	20,877	28	96	✓
DILEKK	WAKAIPGFR	551	579	25,527	18	99	✓
**Peptide HSP-90**	**Peptide PPH-5**						
DRVEVDKNDK	LHkK	624	205	5,506	26	95	
MIKLGLDIGDDEIEDSAVPSSCTAEAK	QKFEAAISTDHDKK	663	147	25,861	73	99	
VEVDKNDKTVK	FEAAISTDHDKkTVAETLDINAMAIEDSYDGPR	624	158	32,007	36	99	
**Peptide GR-LBD**	**Peptide PPH-5**						
QVIAAVKWAK	MYGFEGEVKAK	576	319	22,754	42	85	✓
WAKAIPGFR	MYGFEGEVKAK	579	319	25,375	64	99	✓

Shown are the crosslinked products detected by mass spectrometry for GRLBDm·HSP-90·PPH-5 complexes, using two different search algorithms, xMASS and pLink. Amino acid positions refer to full length, non-tagged proteins as numbered in the Uniprot database. Scan number refers to the MS2 scan with the most hits for the specified peptide. Shown are also the number of hits for this scan, the per cent spectral intensity as well as the agreement between xMASS and pLink.

**Table 2:** Inter-protein crosslinked peptides detected for GRLBDm·HSP-90·FKB-6 complexes.

Peptide 1	Peptide 2	Lys 1	Lys 2	MS2 scan	Hit number in scan	Hit % spectral intensity	Independent analysis
**Peptide HSP-90**	**Peptide GR-LBD**						
APFDLFENKK	ELGKAIAK	326	699	24,087	38	83	✓
DSSTMGYMAAKK	QVIAAVKWAK	601	576	22,389	92	74	✓
APFDLFENKK	MTYIKELGK	326	695	27,231	89	91	✓
SKNSIK	MTYIKELGK	329	695	13,803	77	60	✓
VEKVGVSNR	WAKAIPGFR	555	579	20,880	31	55	✓
DILEKK	WAKAIPGFR	551	579	25,458	18	44	✓
**Peptide HSP-90**	**Peptide FKB-6**						
MKENQTQIYYITGESK	VPATWEMTAEEKLDAAKQAK	455	250	23,025	30	82	
KCMELIDEVAEDKDNFK	MSGEKIDITPKK	402	11	20,677	23	86	✓
DNFKK	YKR	406	278	25,623	18	98	

Shown are the crosslinked products detected by mass spectrometry for GRLBDm·HSP-90· FKB-6 complexes, using the search algorithms, xMASS and pLink. Amino acid positions refer to full length, non-tagged proteins as numbered in the Uniprot database. Scan number refers to the MS2 scan with most hits for the specified peptide. Shown are also the number of hits for this scan, the per cent spectral intensity as well as the agreement of xMASS with the pLink algorithm.

PPH-5 contains a TPR domain located at its N-terminus and a C-terminal αJ subdomain^13,54^. Based on the identified crosslinking sites from the ternary GRLBDm·HSP-90·PPH-5 complex, the phosphatase appears bound to HSP-90 in a similar arrangement, as previously reported for the binary HSP-90·PPH-5 assembly^41^. PPH-5 apparently adopts a head-to-tail topology relative to the chaperone, bringing the N-terminal TPR motifs towards the C-terminus of HSP-90. Crosslinking sites can be identified in the M and CTD domains of HSP-90 (Fig. 3B, Table 1). Importantly, crosslinked products between PPH-5 and GRLBDm can also be found, hinting at an interaction on the chaperone scaffold that possibly sets the basis for the observed cooperativity during complex formation. Lysines 576 and 579 of GRLBDm are linked to lysine 319 that resides in the catalytic domain of the phosphatase.

Performing a similar study with the cofactor FKB-6, crosslinking sites can be identified that help position the cofactor relative to GRLBDm and HSP-90. FKB-6 contains two peptidyl prolyl isomerase (FKBP) domains (FK1, FK2) and a C-terminal tetratricopeptide repeat (TPR) region (Fig. 3D). We identified contacts between the chaperone and FK1 and TPR domains of FKB-6, as summarized in Table 2. The identified crosslinked product that pairs lysine 11 of FKB-6 and lysine 402 of HSP-90, indicates a contact between the FK1 domain of FKB-6 and HSP-90’s M domain (Fig. 3C). It has been defined that the TPR cofactors interact with the Hsp90 machinery via its C terminal MEEVD motif which, lacking lysine residues, cannot be observed as a crosslinking product^55,56^.

We then aimed at confirming the relevance of the FKBP domains to complex formation based on biochemical experiments. To this end we constructed FKB-6 variants that contain the TPR and either the FK1 (FK1-FKB-6) or the FK2 domain (FK2-FKB-6). Sedimentation velocity AUC experiments with FKB-6 truncated constructs revealed that deletion of the FK1 domain maintains robust binding to GRLBDm·HSP-90 whereas, upon removal of FK2, the ternary complex formation, occurs with sharply reduced affinity (Fig. 3E). Thus, both FK domains apparently contribute to the interaction, with the FK2 domain having a stronger influence on the cooperative complex formation.

### Rates of hormone-binding to GR-LBD are modulated by the Hsp90-state

Although Hsp90 is required for hormone binding in vivo, it is known that GRLBD is capable of ligand binding in vitro in the absence of Hsp90^35,38^. It is unclear how the Hsp90 system primes GR for ligand binding, while cofactor-induced effects have been observed^57^. We therefore tested to what extent the observed complexes are competent in hormone binding. A fluorescein-labelled variant of dexamethasone (F-DEX) was used to monitor the kinetic of hormone binding to GRLBDm by fluorescence polarization.

**Figure 4:**
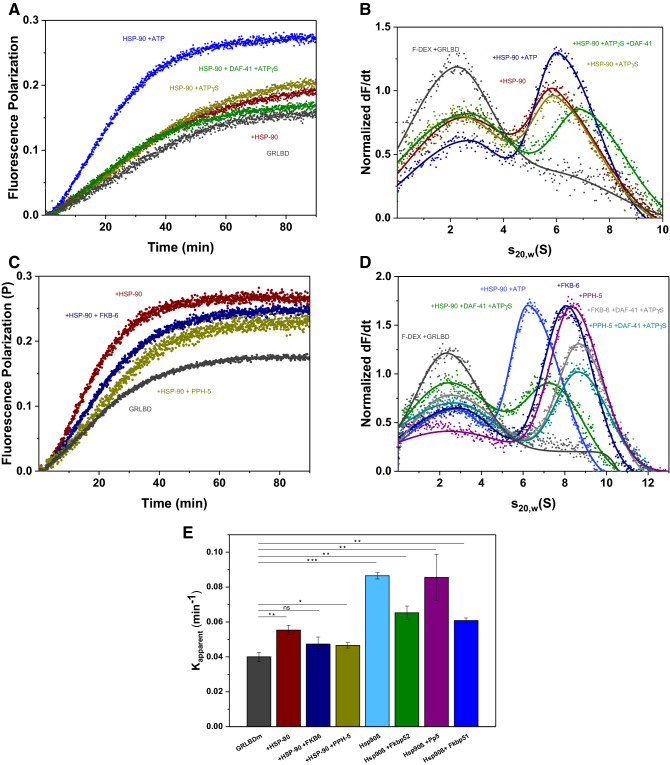
Hormone binding to GRLBDm is modulated by HSP-90, ATP and TPR-cofactors. (**A**) Association kinetics of F-DEX tο GRLBDm (grey) in the presence of *C. elegans* HSP-90 (red) and nucleotides as indicated in the plot. (**B**) Sedimentation velocity analysis of F-DEX bound to GRLBDm (grey) in the presence of *C. elegans* HSP-90 (red) and nucleotides as indicated in the plot. (**C**) Association kinetics of F-DEX tο GRLBDm (grey) in the presence of *C. elegans* HSP-90/ATP (red) and cofactors as indicated in the plot. (**D**) Sedimentation velocity analysis of F-DEX bound to GRLBDm (grey) in the presence of *C. elegans* HSP-90/ATP (red) and cofactors as indicated in the plot. (**E**) Influence of TPR-cofactors on the hormone-binding rates of GRLBDm as determined from fluorescence polarization kinetics. All samples contained HSP-90 and ATP plus the indicated cofactors. Statistical significance was assessed by a two-sample t-test and a level of significance of 0.05. Error bars represent the standard deviation of three independent measurements (n = 3).

We recorded the binding kinetics first in the absence and presence of HSP-90 and then supplemented with ATP, ATPγS, ATPγS/DAF-41. In these reactions ATP presence is crucial to accelerate hormone binding, as any other state of HSP-90 retains binding close to control levels (Fig. 4A). The combination of HSP-90, Cep23/DAF-41 and ATP (Supplementary Fig. S2) seems to stimulate hormone binding to the same extent as HSP-90 and ATP alone, leaving uncertain whether an interaction with Cep23/DAF-41 has taken place, as this interaction is known to be better stabilized by ATPγS and not so much by ATP^52^.

By employing F-DEX in analytical ultracentrifugation measurements, only the F-DEX-bound complexes can be observed, allowing us to confirm the formation of protein complexes from an independent approach. F-DEX alone does not sediment in these experiments, but F-DEX bound to GRLBDm is readily observable at 2.7 S. Complex formation with chaperones then leads to the detection of larger species based on the bound fluorescent hormone. Judging from the reduction in the amount of monomeric F-DEX-bound receptor (Fig. 4B), the HSP-90 complex with hormone-bound GRLBDm is formed most efficiently, if ATP is also present (Fig. 4B). This implies that the rate increase may correlate with the formation of this complex.

Testing the influence of the TPR-cofactors PPH-5 and FKB-6 on the hormone binding to GRLBDm, we find a further modulation of the binding rates compared to GRLBDm·HSP-90·ATP (Fig. 4C,E). The rate of hormone binding is slightly reduced but remains accelerated compared to control reactions. To get clarity on the complex formation, theses assemblies were further investigated by AUC experiments with F-DEX. *C. elegans* ternary complexes with the cofactors were formed as efficiently as with HSP-90/ATP in the case of FKB-6 and slightly more efficiently with PPH-5, as judged from the reduction of monomeric F-DEX-bound receptor at 2.7 S (Fig. 4D). Based on this data, ATP-binding to HSP-90 seems to accelerate the binding of hormone to GRLBDm and slighter modifications to this rate are observable, when cofactors enter the complex (Fig. 4E). Given that residual hormone might be present in the binding pocket, it cannot be excluded that the observed rates represent exchange kinetics and not binding rates. Nevertheless, such acceleration shows that nematode HSP-90 has the ability to influence the hormone binding properties of GRLBDm.

### Hsp90β’s conformation is more restricted in the human Hsp90 system

**Figure 5:**
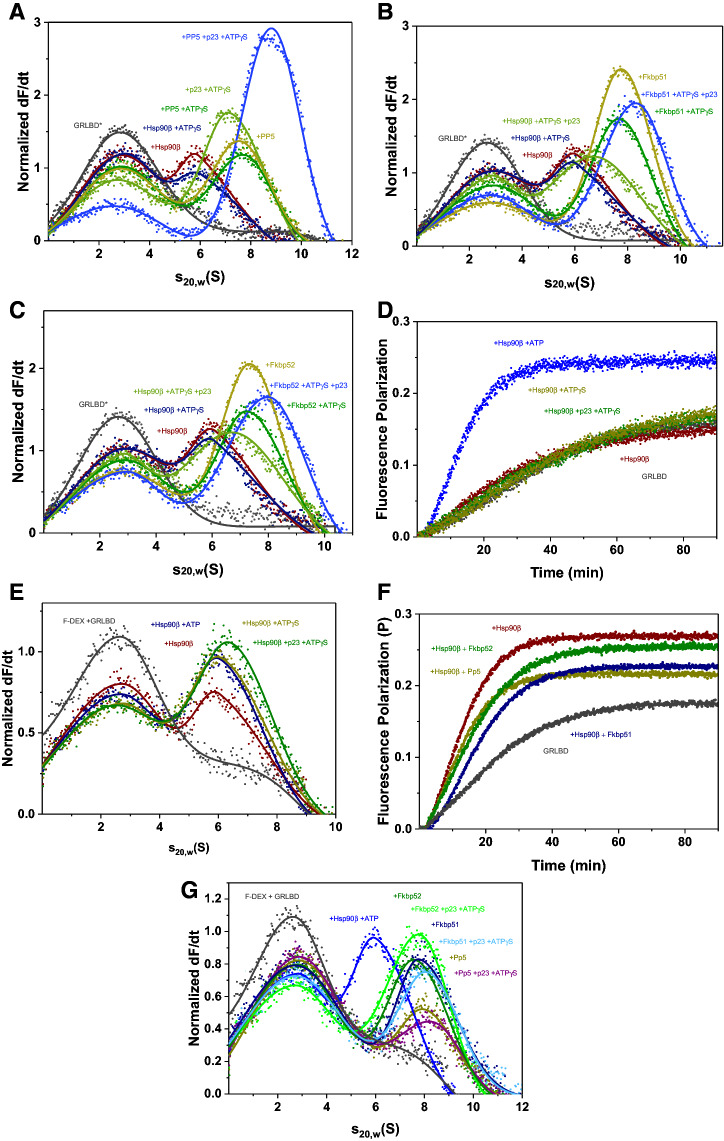
Hsp90β’s conformation is more restricted in the human Hsp90 system. (**A**) Sedimentation velocity AUC analysis of *GRLBDm (grey), *GRLBDm·Hsp90β (red), *GRLBDm·Hsp90β/ATPγS (navy), *GRLBDm·Hsp90β/ATPγS·p23 (light green), *GRLBDm·Hsp90β·Pp5 (yellow), *GRLBDm·Hsp90β/ATPγS·Pp5 (green), *GRLBDm·Hsp90β/ ATPγS·Pp5·p23 (blue) complexes. (**B**) Sedimentation velocity analysis of *GRLBDm·Hsp90β complexes with Fkbp51 and/or p23 set-up accordingly. (**C**) Sedimentation velocity analysis of *GRLBDm·Hsp90β complexes with Fkbp52 and/or p23 set-up accordingly. (**D**) Association kinetics of F-DEX to GRLBDm (grey) in the presence of human Hsp90β (red) and nucleotides as indicated in the plot. (**E**) Sedimentation velocity analysis of F-DEX association to GRLBDm (grey) in the presence of human Hsp90β (red) and nucleotides as indicated in the plot. (**F**) Association kinetics of F-DEX tο GRLBDm (grey) in the presence of human Hsp90β/ATP (red) and cofactors as indicated in the plot. G) Sedimentation velocity analysis of F-DEX association to GRLBDm (grey) in the presence of human Hsp90β/ATP (red) and cofactors as indicated in the plot.

We then aimed at understanding, to what extent the main principles are also conserved in the human system. The complexes assembled from the nematode proteins in this study correspond to those identified for the human system in the 1990s, but only limited in vitro data on the GR-complexes, even on the human ones, are available to date. To compare the two systems, we purified the corresponding human proteins and repeated the experiments described for the nematode system. The human system is known to behave differently from the nematode HSP-90 regarding its ATP-turnover and conformational flexibility^58,59^. We could form GRLBDm containing protein complexes with the protein phosphatase Pp5 and Hsp90β (Fig. 5A). In contrast to the nematode system, no ATPγS induced changes in the human GRLBDm·Hsp90β·Pp5 complex are observable and also no changes in s_20,w_ are observed for the binary GRLBDm·Hsp90β complex after ATPγS addition. Only upon binding of p23 is a strong increase in s_20,w_ observed and the closed form is obviously stabilized (Fig. 5A). Interestingly, we find a cooperative action of ATPγS, p23 and Pp5, which leads to a strong increase in affinity and binding of almost all GRLBDm to the complex. It here seems that a closed Hsp90β complex with the phosphatase Pp5 is very favorable, but only achievable if p23 stabilizes the closed conformation (Fig. 5A).

We then tested both human PPIase homologs, Fkbp51 (Fig. 5B) and Fkbp52 (Fig. 5C). Both PPIases support GR-binding to the chaperone complex, albeit the cooperative effect is reduced compared to the nematode system. In contrast to the nematode FKB-6 complexes, the response of GRLBDm·Hsp90β·Fkbp51/52 complexes to ATPγS is not observable. The presence of ATPγS, however, slightly decreases the affinity for the client. As in the complexes with Pp5, binding of p23 eventually leads to the compaction of the PPIase-containing protein complex (Fig. 5B, C), indicating that ATPγS may not be sufficient to initiate the closing reaction in the human system.

This data shows that the nematode and human chaperone systems share a conserved interaction pattern with the TPR-cofactors involved in the processing of steroid receptors. The human Hsp90β however, shows a reduced ability to form the closed state under the examined conditions and seems to strictly require the cofactor p23 to perform this conformational change.

Similar patterns between the two systems can also be observed for the interaction with the hormone. As in the nematode system, only Hsp90β/ATP can accelerate the binding rate beyond control levels (Fig. 5D). AUC data show that F-DEX-bound protein complexes are formed more efficiently with ATP and also the more compacted ATPγS/p23-bound states of Hsp90β (Fig. 5E). Regarding the influence of human TPR-cofactors, only the hormone-bound quaternary complexes with ATPγS/p23 and either Fkbp51 or Fkbp52 form as efficiently or stronger than the binary GRLBDm·Hsp90β/ATP complex, judging from the reduced F-DEX-bound GRLBDm at 2.7S (Fig. 5G). While the Pp5-containing ternary complex with F-DEX-bound GR-LBDm is weakly associated (Fig. 5G), it is nevertheless able to strongly stimulate the binding rate (Fig. 4E). Thus, like in the nematode system, ATP binding to Hsp90β appears to accelerate the exchange of hormone in GRLBDm and further modifications to the exchange kinetics are observable, when TPR-cofactors enter the complex (Figs. 5F, 4E).

## Discussion

GR·Hsp90 complexes have been studied extensively since the 1980s in cellular lysates of reticulocytes and wheat germ, leading to the identification of Hsp90 as a chaperone bound to GR and also to the description of assemblies involving several Hsp90 co-chaperones^40,60–63^. Much of our current structural understanding of GR complexes in vitro originates from yeast Hsp90 and its cofactors and only recently, structural data on higher eukaryotic species became available^5,35,38,50,64^. The extent and timing of movements performed by Hsp90 and the biochemical contribution of the co-chaperones are still rather unclear, but the recent purification of client proteins makes in vitro studies possible^35–37^.

We here focus on the nematode and human Hsp90 chaperone systems to clarify which conserved principles are observable between the different eukaryotic species. The nematode system contains only one homolog of all major cofactors and a highly conserved Hsp90 protein, which has cellular contact to client proteins of all known Hsp90-client classes. There are at least 200 nuclear receptors in nematodes, but which of them are clients of the Hsp90 machinery is currently unknown. In this study, we used a stabilized GRLBDm mutant of the ligand binding domain of the human GR to model the wild-type protein, which is unstable^65^. The LBD domain of the human GR shows homology to the *C. elegans* nuclear hormone receptors NHR-25, NHR-47 and, FAX-1 that bind so far unknown ligands and to some extent, to the receptor for dafachronic acid, DAF-12.

**Figure 6:**
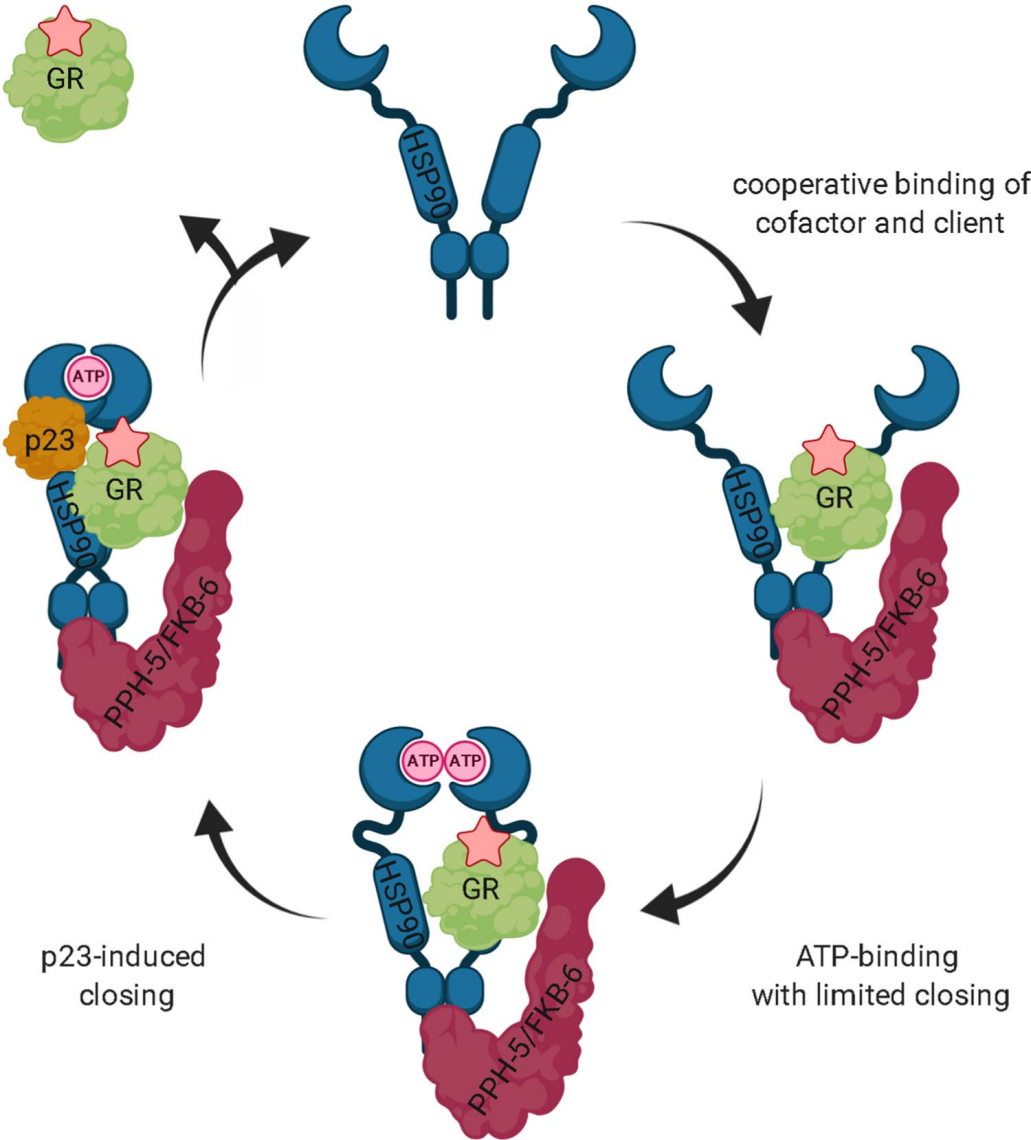
Hypothetical cycle of the *C. elegans* HSP-90 interaction with nematode cofactors and the glucocorticoid receptor LBD. In the case of direct binding of GR to the HSP-90 machinery, cooperative client and cofactor (PPH-5 or FKB-6) binding occurs favoring the indicated stoichiometry. Binding of nucleotide then leads to progression of the cycle towards a more compact state of HSP-90 and to more efficient hormone binding. Upon p23 inclusion in the HSP-90 assemblies, the closed state is stabilized. ATP hydrolysis may then trigger GR and cofactor release. The figure was created with BioRender.com.

Based on our results, *C. elegans* HSP-90 appears to perform its closing reaction with an efficiency in-between yeast and human. Even under conditions where the nucleotide ATPγS is present, formation of the closed, twisted state does not proceed for the isolated HSP-90 or Hsp90β (data not shown). This contrasts with the homologous yeast Hsp82 protein. Instead, the nematode or human Hsp90s remain in an open-like conformation and also the presence of client protein does not induce the closing reaction. The TPR cofactor STI-1 and the ATPase activator AHSA-1 could hardly be detected in GRLBDm·HSP-90 complexes. This is in agreement with experiments performed in yeast^35^. The human homologue of STI-1, Hop, is known to deliver the GRLBD bound to the chaperone Hsp70 to Hsp90 and ATP hydrolysis by Hsp90 is thought to induce the release of Hsp70 and Hop^38^. Thus, STI-1 alone may not be detected as a component of GRLBDm·HSP-90 complexes in the absence of the HSP-70 chaperone. Further, we see that the binary GRLBDm·HSP-90 complex is disrupted once excess STI-1 is added, hinting at a competition for a binding site on HSP-90. Technically, the STI-1·HSP-90 complex cannot be observed by fluorescence AUC with labeled GRLBDm, it is, however, noticeable, since GRLBDm is exclusively observed in the unbound fraction. STI-1 interacts primarily with the C-terminal MEEVD-peptide of Hsp90, but also the M domain, which is likely the binding site that STI-1 and GRLBDm compete for^59,66,67^. For AHSA-1, we also observe competitive binding with GRLBDm, which could be due to the different conformational requirements or overlapping binding sites on the Hsp90 chaperone. In particular, the binding site in the middle domain of the chaperone may be used by both proteins and therefore such ternary complexes may not form efficiently^64,68^.Only in trimeric complexes with the cofactors PPH-5 or FKB-6 does HSP-90 perform nucleotide-induced conformational rearrangements and reach a compacted state. These cofactors also strongly facilitate the binding of GRLBDm to the chaperone. Thus, the nematode HSP-90 cycle seems to be based on cooperative events between TPR-proteins, nucleotide and client protein (Fig. 6).

The cofactor p23 has been thought to stabilize Hsp90-client interactions, which is supported in our experiments with the human chaperone system^36,69^. A recent study, however, shows that p23 stimulates GRLBD dissociation from the chaperone with ATP but not with the non-hydrolysable nucleotides and that it thus, can function as a substrate release factor for Hsp90^70^. For our AUC analysis, we utilize the non-hydrolysable nucleotide ATPγS in an attempt to stabilize the closed states of the client·Hsp90 and client·Hsp90·TPR cofactor complexes in the presence and absence of p23. In this set-up the affinity for Cep23/DAF-41 is rather low. In complexes consisting of the nematode proteins, 0.22 ± 0.05 of the GRLBDm fraction is bound to the chaperone in the presence of ATPγS while 0.28 ± 0.03 is bound when Cep23/DAF-41 is added. In contrast, 0.49 ± 0.05 of GRLBDm is bound to the chaperone in the absence of ATPγS. We therefore, can say with conviction that ATPγS reduces the affinity of HSP-90 to GR, but the further integration of Cep23/DAF-41 may not change the affinity significantly (p = 0.1493). Given that the nematode protein responds much stronger to the binding of ATPγS, it can be assumed that the observed differences between nematode HSP-90 and human Hsp90β relate to the different flexibility and response of the protein to the nucleotide.

**Figure 7:**
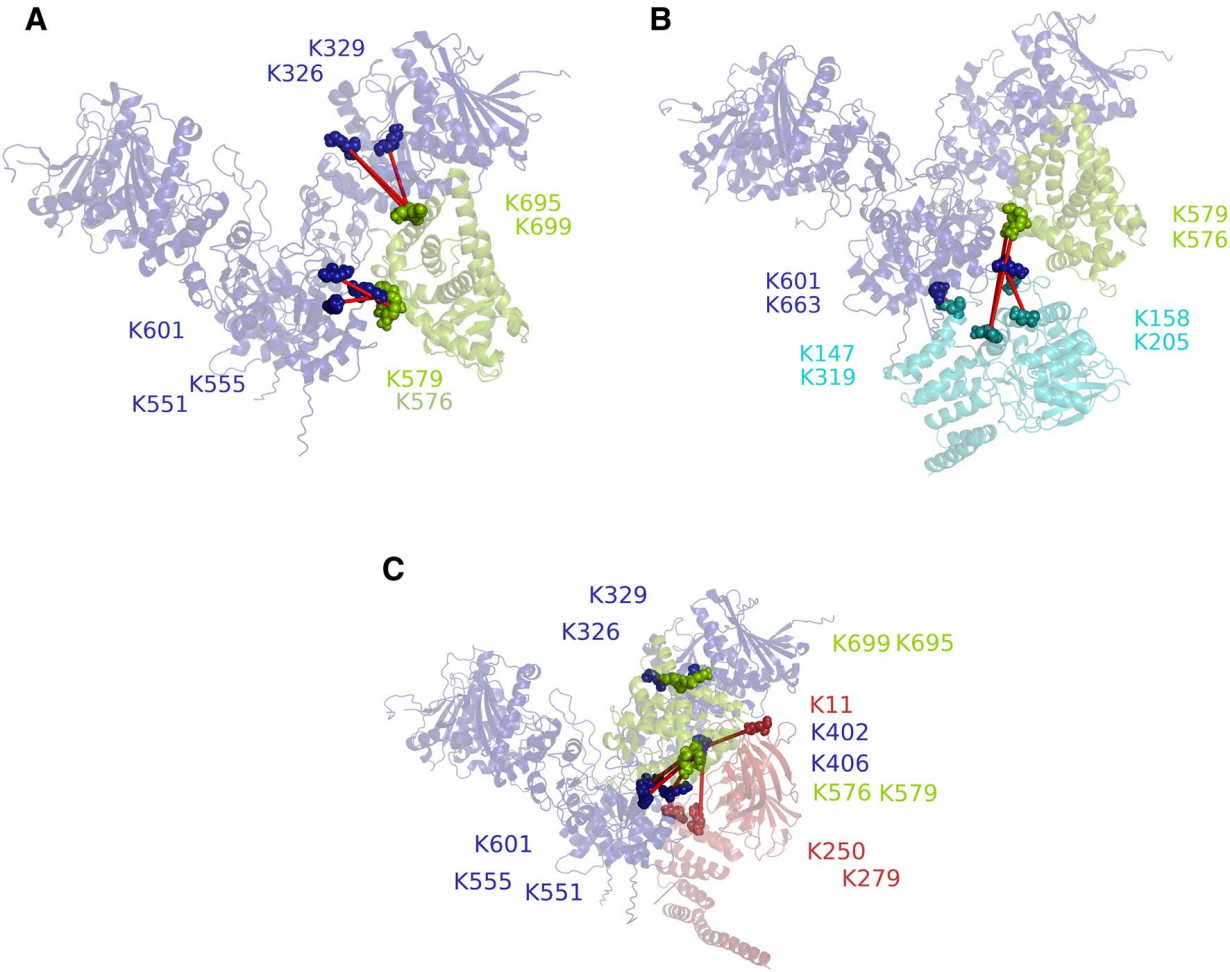
Structural representation of HSP-90 complexes, generated by crosslink guided molecular docking. (**A**) Structural representation of the binary GR-LBDm·HSP-90 complex. HSP-90 is shown in blue, GRLBD in green and spheres represent the crosslinked lysine residues. (**B**) Structural representation of the trimeric GRLBD·HSP-90·PPH-5 complex. HSP-90 is shown in blue, GRLBD in green and PPH-5 in cyan. (**C**) Structural representation of the trimeric GRLBD·HSP-90·FKB-6 complex. HSP-90 is shown in blue, GRLBD in green and FKB-6 in red.

Based on mass spectrometry data, we investigated whether the observed cooperativity in complex formation may originate from contacts between the cofactor and client proteins on the chaperone scaffold. Our structural interpretation for GRLBDm·HSP-90 is in good agreement with the structural model proposed previously for yeast Hsp90 in complex with GRLBDm^35^. Electron microscopy structural studies also report GRLBD being bound to the MD and CTD domains of human Hsp90, as part of a complex with Hsp70, Hop and Hsp90^38^. This binding site is also in line with the client-binding region of *E. coli* Hsp90 described by Genest et al.^71^. Our results on the GRLBDm·HSP-90·FKB-6 topology are consistent with recent findings regarding the human Fkbp51^72^. This study also postulates a stepwise interaction with Hsp90, with affinities decreasing in the order TPR > FK2 > FK1. In the case of PPH-5, the arrangement of the cofactor in GRLBDm·HSP-90·PPH-5 complexes is similar to our previous study on the binary HSP-90·PPH-5 complex^41^. To illustrate these topologies, we used the identified crosslinked peptides in docking calculations with HADDOCK and obtained structural models for the dimeric GRLBDm·HSP-90 complex (Fig. 7A) as well as the trimeric complexes with the two TPR cofactors (Fig. 7B, C)^73,74^. These calculations bring both cofactors’ catalytic domain in close proximity to the client (Fig. 7B, C), which may be setting the basis for the observed cooperativity. The client may then be accessible to undergo transformations towards dephosphorylation and receptor maturation.

The cooperative complex formation and hormone binding patterns seem similar in the nematode and human Hsp90 systems, albeit the persistence of the “open” state is stronger for the human Hsp90β protein. Here, Fkbp-containing and Pp5-containing complexes appear unable to perform the closing reaction induced by ATPγS and only with the support of p23 are these rearrangements possible and lead to the progression of the Hsp90 cycle. This data highlights that, despite the conserved features of the cofactors, the different degree of flexibility within the Hsp90 protein from the two organisms influences the principles during chaperone·cofactor·client complex formation.

## Material and methods

### Protein expression and purification

Proteins were expressed in *Escherichia coli* BL21 (DE3) cells, utilizing pET28 plasmids as expression vectors to generate proteins containing an N-terminal His_6_-tag. Bacterial cultures were grown at 37 °C to an OD_600_ of 0.6 and expression was induced by addition of 0.5 mM IPTG. Cells were incubated overnight at room temperature and harvested by centrifugation with 7,000 rpm for 15 min at 4 °C. Cell pellets were resuspended in 40 mM HEPES/KOH, 150 mM KCl, pH 7.5 supplemented with protease inhibitor (Serva, Heidelberg, Germany) and DNAseI (Serva, Heidelberg, Germany) and were mechanically disrupted by a hydraulic press (Constant Systems Ltd., Daventry, UK). Cleared lysate was applied onto a HisTrap FF 5 ml column (GE Healthcare, Chicago, USA) and elution was induced by 300 mM imidazole. Protein containing fractions were diluted, applied onto a Resource Q (GE Healthcare, Chicago, USA) column and eluted in a salt gradient. Proteins were then purified to homogeneity on a Superdex 75 or Superdex 200 size-exclusion column (GE Healthcare, Chicago, USA) equilibrated in storage buffer (40 mM HEPES/KOH, 150 mM KCl, 0.5 mM, pH 7.5). Proteins purified according to this procedure, such as nematode HSP-90, Cep23/DAF-41, AHSA-1, STI-1, FKB-6, PPH-5 or the human proteins p23, Hsp90β, Fkbp51, Fkbp52 and Pp5 were shock-frozen in liquid nitrogen and stored at − 80 °C. Identity and purity were assessed by MALDI-TOF mass spectrometry (Bruker, Bremen, Germany) and SDS-PAGE (data not shown). GRLBDm, the ligand-binding domain of GR stabilized by mutagenesis (F602S/A605V/V702A/E705G/M752T), was expressed at 18 °C overnight in ZYM-5052 media supplied with 250 μM dexamethasone (Serva, Heidelberg, Germany) and purification was performed as described previously^35,65^.

### Fluorescence-labelling of GRLBDm

0.1 mg ATTO 488 maleimide (ATTO-Tech, Singen, Germany) dissolved in DMSO was added to 1 mg of GRLBDm with a final DMSO concentration of 1%. The reaction was carried out for one hour at room temperature and was quenched with 100 mM DTT. Free label was removed by dialysis against 25 mM Tris, 100 mM NaCl, 50 μΜ dexamethasone and 0.5 mM DTT.

### Analytical ultracentrifugation (AUC)

Sedimentation analysis of ATTO 488-labelled GRLBDm (*GRLBD) was performed with a ProteomeLab Beckman XL-A analytical ultracentrifuge (Beckman Coulter, Brea CA, USA) with an AVIV fluorescence detection system (Aviv Biomedical Inc., Lakewood CA, USA). Ultracentrifugation was carried out in 20 mM HEPES, 20 mM KCl, 5 mM MgCl_2_, 50 μΜ dexamethasone, pH 7.5^2,35,75^. Experiments contained 600 nM of labelled GRLBDm (*GRLBD) and 3 μM of the unlabelled chaperones and cofactors of interest. Nucleotides were added at a concentration of 2 mM. GRLBD alone showed a weak tendency to aggregate, which was absent once Hsp90 and cofactors were present. This tendency is visible in the dc/dt plots as an extension towards higher s-values and a deviation from the symmetric peak shape. AUC-experiments monitoring F-DEX (Thermo Fischer Scientific, Bremen, Germany) utilized 400 nM F-DEX and 3 μM of the unlabelled proteins of interest. These measurements were carried out in 20 mM HEPES, 20 mM KCl, 5 mM MgCl_2_, pH 7.5. Centrifugation was performed at 42,000 rpm at 20 °C. Data analysis was performed by calculating differences between scans from a selected time range and averaging over several of these differentials. The dF/dt data was then normalized against the initial fluorescence intensity. To ensure comparable sample handling, plots were generated from samples measured in the same experiment with automated data processing in the in-house software diffUZ^41^. s_20,w_ values were derived from a bi-Gaussian fitting of the dF/dt plots and the error is based on the standard deviation of this approach plus an additional contribution from performing the meniscus picking procedure. In cases were trends were not always conserved between replicate analyses, all replicates were used to calculate significance levels for potential effects. Significance was achieved, if p-values from a Student’s t-test were below 0.01. In general repeatability of AUC results decreased if three or more weakly binding components were added at concentrations below saturation levels.

### Fluorescence polarization

Hormone binding kinetics were monitored by fluorescence polarization on a Jasco FP-8500 fluorescence spectrometer (Jasco, Groß-Umstadt, Germany) equipped with polarizers. 1 µM apo-GRLBDm, after extensive dialysis to remove dexamethasone as described, was added to various chaperone mixtures with a chaperone and cofactor concentration of 3 µM^35,38^. Binding kinetics to 50 nM fluorescently labelled dexamethasone (F-DEX, Thermo Fischer Scientific, Bremen, Germany) were recorded at 20 °C in 20 mM HEPES, 20 mM KCl, 5 mM MgCl_2_, 2 mM ATP, pH 7.5. Hormone binding rates were determined by fitting association kinetics to exponential models and the error bars represent the standard deviation of three independent measurements.

### Crosslinking experiments

The crosslinking reactions were carried out in 20 mM HEPES, 20 mM KCl, 5 mM MgCl_2_, pH 7.5 for ten minutes at room temperature, using the bis-sulfo-succinimidyl-suberate crosslinker (BS^3^-H12/D12) (Creative Molecules, Scottsdale, USA) in a 50-fold excess over protein, as described previously^41^. The crosslinking reaction was quenched by the addition of 5 × Laemmli buffer. Samples were analysed on SERVAGel Neutral pH 7.4 gradient gels (Serva, Heidelberg, Germany) and bands representing the crosslinked species were excised.

### In-gel digestion

Protein bands were washed and destained by three times alternating 10 min treatments with buffer A (10 mM ammoniumhydrogencarbonate, pH 8.3) and buffer B [buffer A:100% acetonitrile from Merck KGaA, Darmstadt, Germany in a ratio of 50:50 (v/v)], as described previously^76,77^. After the second incubation with 50 mM ammonium bicarbonate, samples were treated with 50 μl 10 mM DTT (AppliChem GmbH, Darmstadt, Germany) for 1 h at 56 °C and with 50 μl 50 mM IAA (Merck KGaA, Darmstadt, Germany) for 45 min at room temperature before the destaining protocol was continued. Finally, gel pieces were dried in a vacuum concentrator (RVC2-25CD plus, Martin Christ Gefriertrocknungsanlagen, Osterode am Harz, Germany). Digestion was initiated by adding 8 μl of trypsin solution (0.015 μg/μl, Serva, Heidelberg, Germany) and was performed overnight. The digestion was stopped, and peptides were eluted by incubating the gel pieces two times for 15 min with 30 μl of a 1:1 solution containing 100% acetonitrile and 0.1% (v/v) TFA (Merck KGaA, Darmstadt, Germany) in an ice-cooled ultrasonic bath. Samples were dried in a vacuum concentrator and resuspended in 20 μl 0.1% (v/v) TFA. Afterwards, the peptide concentration was determined by amino acid analysis (AAA) as described by Plum et al.^78^.

### NanoLC-ESI–MS/MS

200 ng tryptically digested samples were measured by nanoLC-ESI–MS/MS as described previously^79^. An UliMate 3,000 RSLC nano LC system (Thermo Fischer Scientific, Bremen, Germany) was utilized for nano HPLC analysis using the following solvent system: (A) 0.1% FA; (B) 84% ACN, 0.1% FA. Samples were initially loaded on a trap column (Thermo Fischer Scientific, 100 μm × 2 cm, particle size 5 μm, pore size 100 Å, C18) with a flow rate of 30 μl/min with 0.1% TFA. After sample concentration and washing, the trap column was serially connected with an analytical C18 column (Thermo Fischer Scientific, 75 μ m × 50 cm, particle size 2 μm, pore size 100 Å), and the peptides were separated with a flow rate of 400 nl/min using a solvent gradient of 4% to 40% B for 95 min at 60 °C. After each sample measurement, 1 h of column washing was performed for equilibration. The HPLC system was on-line connected to the nano-electrospray ionization source of a Q Exactive HF mass spectrometer (Thermo Fischer Scientific, Bremen, Germany). The mass spectrometer was operated in a data-dependent mode with the spray voltage set to 1,600 V in positive mode and a capillary temperature of 275 °C. Full scan MS spectra (mass range 350–2000 m/z) were acquired in the Orbitrap analyzer at a mass resolution of 60,000. The twenty most intensive ions per spectrum were subsequently fragmented using collision-induced dissociation (35% normalized collision energy) and scanned in the linear ion trap. The m/z values triggering MS/MS were set on a dynamic exclusion list for 30 s.

### Identification of crosslinked peptides

Initial data analysis was performed with MaxQuant 1.5 to obtain lists of all peptides and peaks from the raw data files^80^. These tables were then searched with xMASS as described^41^, yielding crosslinks in four categories with either crosslinker attached at one (Type 1) or both ends (Type 2) on one peptide, crosslinker bridging peptides from the same protein (Type 3) or crosslinker bridging peptides from different proteins (Type 4). These crosslinked structures could be filtered for the same intensity of the peaks separated by 12.07 Da, co-elution from the column, fragmentation spectrum in MS2 and other potential solutions with a similar score. In parallel, the software pLink was used to search the same datasets with the default parameter settings^53^.

### Molecular modelling and Docking calculations

A homology model for the open HSP-90 conformation was generated with ADP-bound HtpG from *E. coli* (PDB 2IOP) as a template, using the Chimera interface to MODELLER^50,81–83^. FKB-6 and PPH-5 were modelled based on human FKBP51 (PDB 5NJX) and rat PP5 (PDB 4JA9) respectively^84,85^. For GR-LBD we used the structure solved by Hemmerling et al. (PDB 5NFP)^86^. The validity of each crosslinked pair was first confirmed using the algorithm DisVis^87,88^. Docking was performed in the expert interface of HADDOCK^73^. Lysine residues identified in crosslinking products were defined as active residues, enforcing a distance restraint of 30 Å between their Cb-atoms, to direct the docking calculations. Structures from the best binary solutions were used to proceed with docking the third protein and assemble the trimeric complexes.

## Supplementary information

Supplementary Figures

〹

## Data Availability

The datasets generated during and/or analysed during the current study are available from the corresponding author on reasonable request.
