# Supplementary material for: Glucocorticoid receptor complexes form cooperatively with the Hsp90 co-chaperones Pp5 and FKBPs
Source: Sci Rep. 2020 Jul 1;10:10733. doi: 10.1038/s41598-020-67645-8 (PMC7329908; doi:10.1038/s41598-020-67645-8)
Supplement: Supplementary file 1 — Supplementary Figures [file 41598_2020_67645_MOESM1_ESM.docx]

# Glucocorticoid receptor complexes form cooperatively with the Hsp90 co-chaperones Pp5 and FKBPs

**Anna Kaziales^1^, Katalin Barkovits^2^, Katrin Marcus^2^ and Klaus Richter^1^***

^1^ Center for Integrated Protein Science Munich at the Department of Chemistry, Technische Universität München, Lichtenbergstr. 4, 85748 Garching

^2^Medical Proteome Center, Ruhr University Bochum, Bochum, Germany.

*To whom correspondence should be addressed: Klaus Richter, Department of Chemistry, Technische Universität München, Lichtenbergstr. 4, 85748 Garching, [klaus.richter@richterlab.de](mailto:klaus.richter@richterlab.de), <Tel:+49-8928913342>

**Supplementary Figures**

**Supplementary Figure S1.**

**
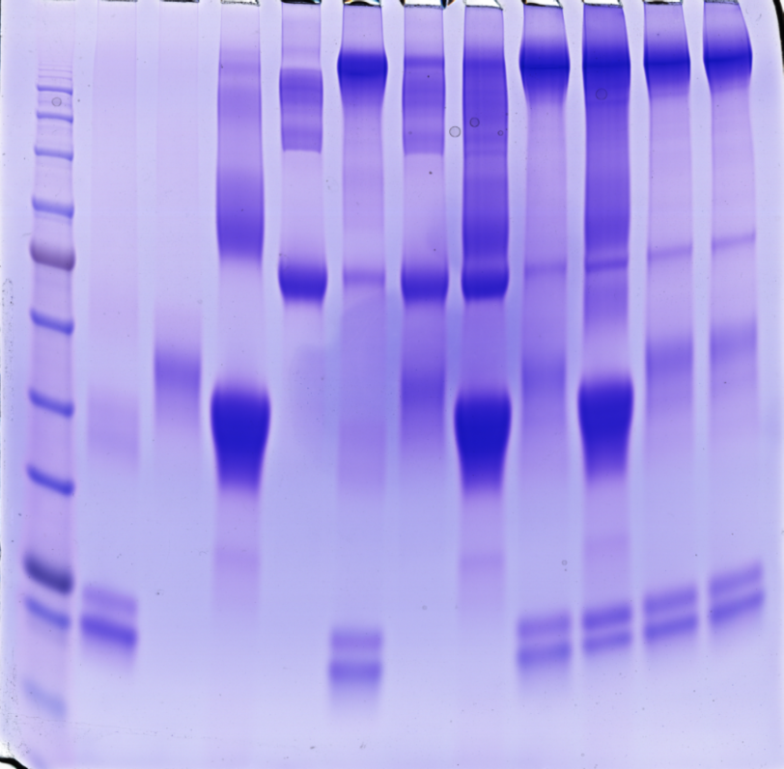
**

**Supplementary Figure S1.** Uncut gel image corresponding to Figure 3A.

**Supplementary Figure S2.**

**
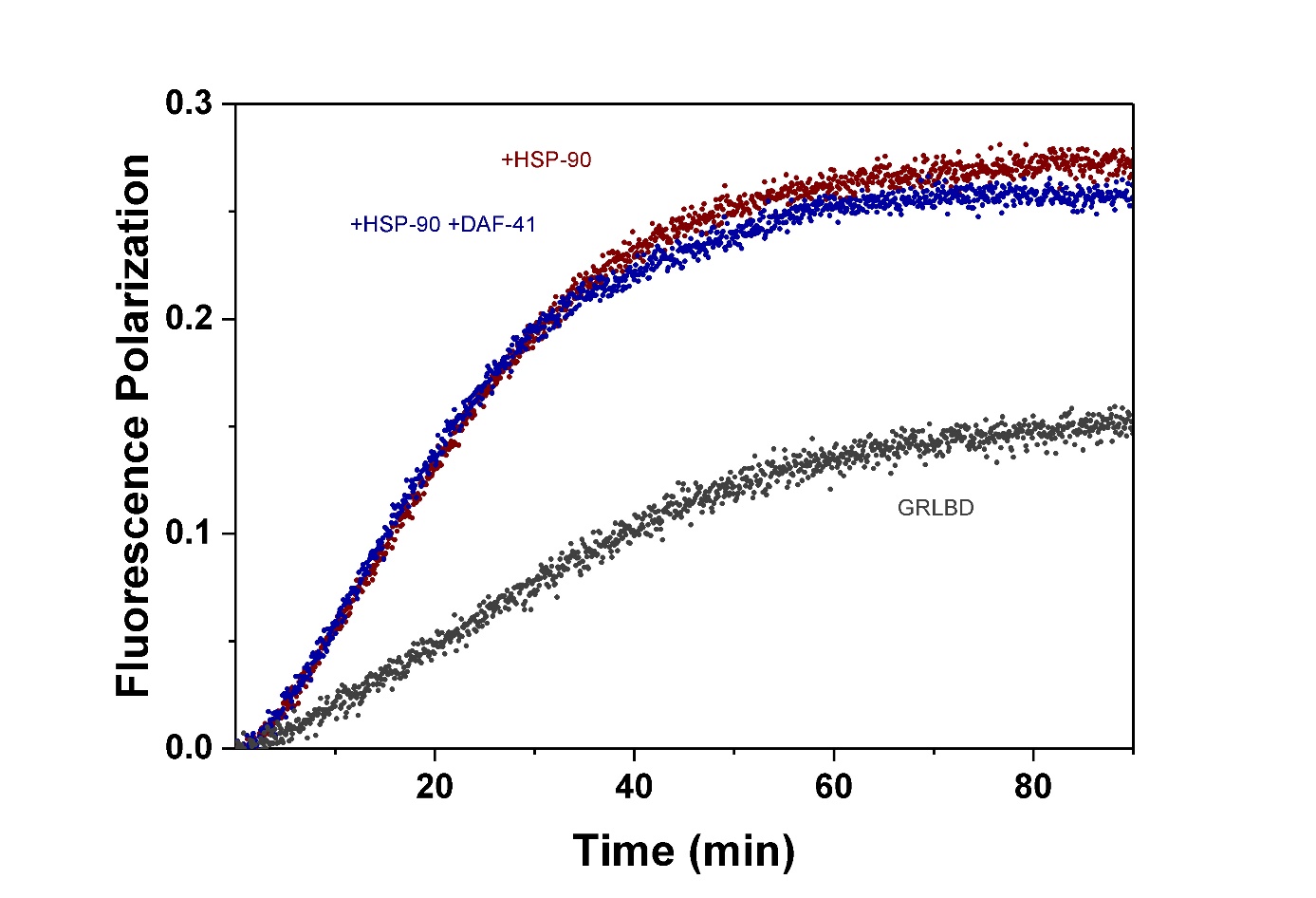
**

**Supplementary Figure S2.** Association kinetics of F-DEX tο GRLBDm (grey) in the presence of *C. elegans* HSP-90 (red) and ATP and in the presence of HSP-90, DAF-41 and ATP (navy). No significant change was observed in the presence of DAF-41.
